# Comprehensive Analysis of the Prognostic Significance of Hsa-miR-100-5p and Its Related Gene Signature in Stomach Adenocarcinoma

**DOI:** 10.3389/fcell.2021.736274

**Published:** 2021-09-17

**Authors:** Gaoming Wang, Ludi Yang, Miao Hu, Renhao Hu, Yongkun Wang, Bo Chen, Xiaohua Jiang, Ran Cui

**Affiliations:** ^1^Department of Hepatopancreatobiliary Surgery, Shanghai East Hospital, School of Medicine, Tongji University, Shanghai, China; ^2^Department of Ophthalmology, Ninth People’s Hospital, Shanghai, China

**Keywords:** stomach adenocarcinoma, hsa-miR-100-5p, prognosis, LASSO, WGCNA

## Abstract

Stomach adenocarcinoma (STAD) is one of the most common cancers in the world. However, the prognosis of STAD remains poor, and the therapeutic effect of chemotherapy and immunotherapy varies from person to person. MicroRNAs (miRNAs) play vital roles in tumor development and metastasis and can be used for cancer diagnosis and prognosis. In this study, hsa-miR-100-5p was identified as the only dysregulated miRNA in STAD samples through an analysis of three miRNA expression matrices. A weighted gene co-expression network analysis (WGCNA) was performed to select hsa-miR-100-5p-related genes. A least absolute shrinkage and selection operator (LASSO) Cox regression analysis was performed to establish a miR-100-5p-related prognostic signature. Kaplan–Meier analyses, nomograms, and univariate and multivariate Cox regression analyses were used to evaluate the prognostic signature, which was subsequently identified as an independent risk factor for STAD patients. We investigated the tumor immune environment between low- and high-risk groups and found that, among component types, M2 macrophages contributed the most to the difference between these groups. A drug sensitivity analysis suggested that patients with high-risk scores may be more sensitive to docetaxel and cisplatin chemotherapy and that patients in the low-risk group may be more likely to benefit from immunotherapy. Finally, external cohorts were evaluated to validate the robustness of the prognostic signature. In summary, this study may provide new ideas for developing more individualized therapeutic strategies for STAD patients.

## Introduction

Gastric cancer is the fifth most common cancer and the third most common cause of cancer death in the world (Smyth et al., 2020). Approximately 90%–95% of gastric cancers are adenocarcinoma. Surgery is the only curative option, and recurrence is common, even after complete resection (Johnston and Beckman, 2019). For most stomach adenocarcinoma (STAD) patients, the diagnosis is received after the most opportune surgical window has passed (Song et al., 2017). Moreover, the prognosis of advanced STAD remains poor despite the use of chemotherapy and biological agents (Coutzac et al., 2019). Chemotherapy and immunotherapy are valid, revolutionary approaches for treating patients with cancer. However, the therapeutic effects are still limited and vary from person to person because of drug resistance or low sensitivity. Hence, it is essential to identify novel molecular biomarkers to improve the prognosis, prediction of recurrence, and treatment response of STAD.

MicroRNAs (miRNAs) are small non-coding RNAs that regulate gene expression by recognizing cognate sequences and interfering with transcriptional, translational, or epigenetic processes. Previous studies have revealed that many miRNAs are dysregulated in cancers and play important roles in tumor proliferation, apoptosis, metastasis, and angiogenesis (Lee and Dutta, 2009). In addition, miRNAs are considered potential biomarkers and therapeutic targets for gastric cancer (Shin and Chu, 2014).

The tumor immune microenvironment (TIME) is the cellular milieu where the tumor is located and is composed of the extracellular matrix, blood and/or lymphatic vessels, fibroblasts, immune cells, and inflammatory cells (Quail and Joyce, 2013). Cross talk between infiltrating immune cells and cancer cells ultimately leads to an environment that fosters tumor development and invasion (Hinshaw and Shevde, 2019). Therapeutics targeting predominant components of the TIME may increase the likelihood of improving patient outcomes. Therefore, the main components and regulatory mechanism of the TIME in STAD need to be further investigated.

**FIGURE 1:**
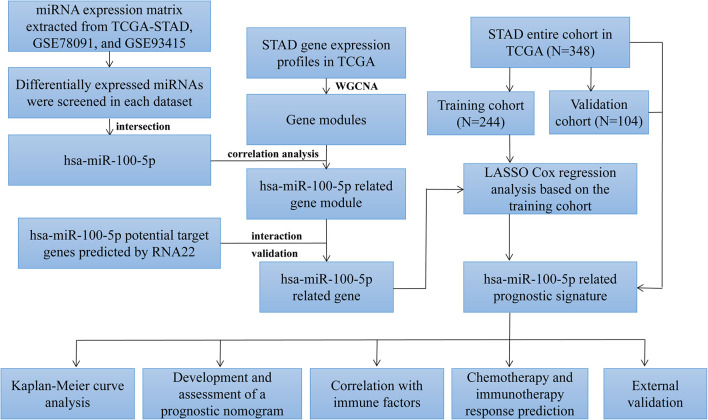
Flowchart of this study.

In the present study (Figure 1), hsa-miR-100-5p was identified as the only miRNA aberrantly expressed in STAD samples. Comprehensive analyses were performed to explore the prognostic capacity of this potential marker for predicting the overall survival of STAD patients. Subsequently, hsa-miR-100-5p-related genes were selected, and a prognostic signature was established. A nomogram featuring integrated clinical features was developed to predict the overall survival (OS) of STAD patients and improve prognostic risk stratification. Next, the potential regulatory mechanism resulting in the difference in the TIME between low- and high-risk groups was further investigated. The sensitivity of the patients to chemotherapy and immunotherapy was evaluated. Finally, external cohorts were evaluated to validate the robustness of the hsa-miR-100-5p-related prognostic signature. This study may assist in improving therapeutic strategies for STAD patients.

## Materials and Methods

### Data Collection and Preparation

The miRNA expression profiles of STAD samples and adjacent normal tissues were acquired from The Cancer Genome Atlas (TCGA) database (TCGA-STAD^1^) and Gene Expression Omnibus (GEO) database [GSE78091, GSE93415 (Sierzega et al., 2017)^2^ ]. The gene expression profiles and corresponding clinical information of STAD patients were obtained from the TCGA and GEO databases [TCGA-STAD, GSE26253 (Lee et al., 2014), GSE15460 (Subhash et al., 2018), and GSE84437 (Yoon et al., 2020), Supplementary Table 1]. Counts and fragments per kilobase million (FPKM) of STAD samples were downloaded from TCGA database on April 13, 2021, and FPKM values were subsequently normalized as transcripts per kilobase millions (TPMs) and transformed as log2(TPM + 1). For samples from the GEO database, the gene expression level was transformed by log2 using the script from GEO2R (Barrett et al., 2013).

### Differential Expression Analysis

Differentially expressed miRNAs (DEMs) between STAD samples and adjacent normal tissues obtained from TCGA database were screened using the R package “DESeq2” (Love et al., 2014) based on the count matrix with thresholds of —log2FoldChange— > 1.0 and adjusted *p*-values (padj) < 0.05. GEO2R, an R-based web application that helps users analyze GEO data, was utilized to screen the DEMs between tumor and normal samples from GSE78091 and GSE93415 with the same conditions (Barrett et al., 2013).

### Identification and Validation of Hsa-miR-100-5p-Related Genes

Weighted gene co-expression network analysis (WGCNA) was used to establish a scale-free co-expression network using R package “WGCNA” to identify the most correlated gene module with hsa-miR-100-5p (Langfelder and Horvath, 2008). RNA22, one of the popular miRNA target prediction algorithms, was utilized to get the potential target genes of hsa-miR-100-5p (Loher and Rigoutsos, 2012). The hsa-miR-100-5p-related candidate genes were identified from the intersection of “hsa-miR-100-5p-related module” and “hsa-miR-100-5p targets.” To validate the candidate genes as robust hsa-miR-100-5p-related genes, STAD samples with both miRNA and gene expression profiles were assigned into two clusters by employing R package “ConsensusClusterPlus” and were subsequently analyzed using principal component analysis (PCA) and uniform manifold approximation and projection (UMAP) (Dorrity et al., 2020).

### Construction and Evaluation of Hsa-miR-100-5p-Related Signatures

A total of 348 STAD patients with the corresponding clinical information retrieved from the TCGA database were randomly assigned into a training cohort (244 patients) and a validation cohort (104 patients) at a 7:3 ratio by using the R package “caret.” The baseline clinical characteristics of the two cohorts are summarized in Supplementary Table 2. The validation cohort and the entire cohort were used as the internal validation cohorts. Besides, the cohorts obtained from GSE26253 and GSE84437 were used as the external validation cohorts. The least absolute shrinkage and selection operator (LASSO) Cox regression analysis was performed based on the training cohort and hsa-miR-100-5p-related genes through R package “glmnet” (Engebretsen and Bohlin, 2019). Signatures were screened by selecting the optimal penalty parameter *λ* correlated with the minimum 10-fold cross-validation. The coefficients (*β*) were extracted from the LASSO Cox regression algorithm and the risk score of each patient was calculated by using the following formula: risk score = expression of gene_1_ × *β*_gene1_ + expression of gene_2_ × *β*_gene2_ + …expression of gene*_*n*_* × *β*_gene_*_*n*_*. Patients in each cohort were divided into high- and low-risk groups according to the optimal risk score cutoff value determined by R package “survminer.” Kaplan–Meier (K-M) curves were plotted to demonstrate the survival difference between the high- and low-risk groups. Receiver operating characteristic (ROC) curves and the area under the curve (AUC) were used to evaluate the predictive performance of gene signatures.

### Development and Assessment of a Prognostic Nomogram

A nomogram interacting with the risk score and clinical factors was established based on multivariate Cox regression analysis using R packages “survival” and “regplot” (Iasonos et al., 2008; Zhang et al., 2018). Calibration curves were adopted to evaluate the accuracy of the nomogram in predicting 3- and 5-year survival rates of STAD patients. Survival net benefits of each variable were estimated with decision curve analysis (DCA) using R package “ggDCA” (Vickers and Elkin, 2006).

### Functional Enrichment Analysis

The R package “clusterProfiler” was utilized to perform the Gene Ontology (GO) enrichment analysis, Kyoto Encyclopedia of Genes and Genomes (KEGG) pathway analysis, and gene set enrichment analysis (GSEA) (Yu et al., 2012). The collection of annotated gene sets in “h.all.v7.4.symbols.gmt” in Molecular Signatures Database (MSigDB^3^) was chosen as the reference gene sets in GSEA (Liberzon et al., 2015). The results of enrichment analysis were visualized using R package “enrichplot” and adjusted *p*-value < 0.05 was chosen as the cutoff criterion.

### Comprehensive Analysis of Immune Infiltration of the Tumor Microenvironment

The immune scores of STAD samples were generated to estimate the levels of infiltrating immune cells by using “Estimation of STromal and Immune cells in MAlignant Tumours using Expression data” (ESTIMATE) algorithm (Yoshihara et al., 2013). The fraction of immune cell types for each sample was quantified through TIMER (Li et al., 2017), quanTIseq (Finotello et al., 2019), and CIBERSORT (Newman et al., 2019) algorithms.

### Estimation of Drug Sensitivity and Response to Immune Checkpoint Blockade

The sensitivity of each patient to chemotherapy drugs was estimated and the half maximal inhibitory concentration (IC50) was quantified using R package “pRRophetic” (Geeleher et al., 2014). Tumor mutation burden (TMB) for each patient was calculated *via* the R package “maftools” (Mayakonda et al., 2018). The response to immune checkpoint blockade (ICB) was predicted by the Tumor Immune Dysfunction and Exclusion (TIDE) algorithm using python (version 3.8.6) script (Jiang et al., 2018).

### Biospecimens and Real-Time Polymerase Chain Reaction Analysis of the miRNAs

A total of seven pairs of gastric tumors and adjacent normal tissues were obtained from Shanghai East Hospital Biobank. All patients had signed informed consent for donating their specimens to Shanghai East Hospital Biobank. Total RNA was extracted *via* TRIpure Total RNA Extraction Reagent (ELK Biotechnology, Hubei, China, EP013) according to the manufacturer’s instructions, following reversed transcribed using EntiLink^TM^ 1st Strand cDNA Synthesis Kit (ELK Biotechnology, EQ003). Quantitative real-time polymerase chain reaction (qRT-PCR) was performed using QuantStudio 6 Flex (Life Technologies, Carlsbad, CA, United States) and EnTurbo^TM^ SYBR Green PCR SuperMix (ELK Biotechnology, EQ001). The primer sequences of hsa-miR-100-5p and small nuclear RNA U6 are listed in Supplementary Table 3. The relative expression level was calculated using the 2^–ΔΔCT^ method and normalized to U6 as the internal control.

### Statistical Analysis

Statistical tests were conducted through R (version 4.0.3). Comparisons between two groups were performed *via* Wilcoxon rank-sum test. Categorical variables were compared with chi-square tests in the training and validation cohorts. Kaplan–Meier curves for overall survival were generated and the difference between groups was compared with the log-rank test. Univariate and multivariate Cox regression analyses were performed to determine the independent prognostic value of hsa-miR-100-5p or the risk score-integrated clinical characteristics. Correlation analysis was conducted using the Pearson correlation test. The random-effects meta-analysis model was used to calculate a pooled hazard ratio (HR) *via* the R package “meta.” *p* < 0.05 was considered statistically significant.

## Results

### Hsa-miR-100-5p Was Downregulated in Stomach Adenocarcinoma Samples and Was Associated With the Prognosis of Stomach Adenocarcinoma Patients

**FIGURE 2:**
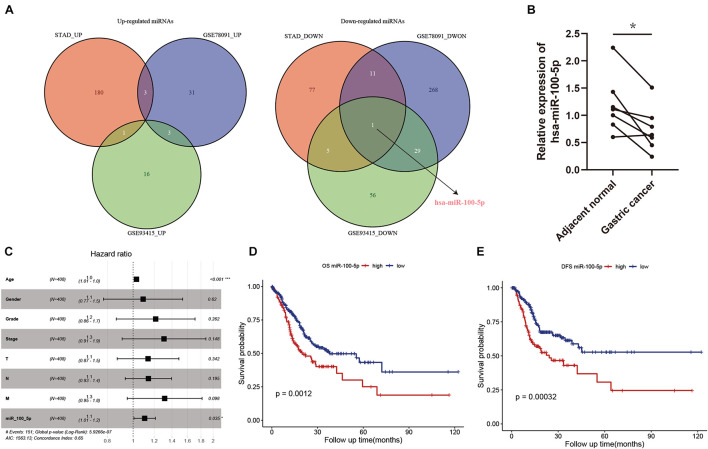
The abnormal expression and prognostic value of hsa-miR-100-5p in stomach adenocarcinoma (STAD). **(A)** Venn diagrams of differentially expressed miRNAs. **(B)** The relative expression level of hsa-miR-100-5p was downregulated in gastric cancer compared with adjacent normal tissues. **(C)** Multivariate Cox analysis of miR-100-5p and clinical features. **(D,E)** Kaplan–Meier survival analyses of miR-100-5p in STAD patients on overall survival **(D)** and disease-free survival **(E)**. *p < 0.05; ***p < 0.001.

In our present study, we identified differentially expressed miRNAs in STAD samples and adjacent normal tissues. Compared with normal tissues, 184, 37, and 20 upregulated miRNAs and 94, 309, and 91 downregulated miRNAs were identified in the STAD samples based on the miRNA expression profiles extracted from the TCGA-STAD, GSE78091, and GSE93415 datasets, respectively. We selected the overlapping miRNAs with the same expression pattern. As the Venn diagram shows, hsa-miR-100-5p was the only miRNA that exhibited a downregulated expression pattern in the different expression matrices (Figure 2A). We further validated the results above by using biospecimens and found that the relative expression of hsa-miR-100-5p was significantly lower in gastric tumors than that in adjacent normal tissues (Figure 2B). Then, we performed Cox analysis to explore connections between hsa-miR-100-5p expression and OS and other multivariable clinical features in STAD patients. As shown in Supplementary Table 4, the univariate analysis revealed some related factors: age (HR=1.02, *p* = 0.017), pathological stage (HR = 1.62, *p*<0.001), T stage (HR = 1.36, *p* = 0.002), N stage (HR = 1.35, *p*<0.001), M stage (HR = 1.56, *p*<0.001), and hsa-miR-100-5p expression (HR = 1.12, *p* = 0.007). The multivariate analysis revealed that hsa-miR-100-5p expression (HR = 1.11, *p* = 0.035) was an independent risk factor for the overall survival of STAD patients (Figure 2C). According to the optimal cutoff point, STAD patients were assigned to low and high hsa-miR-100-5p expression groups. Kaplan–Meier curves showed that patients who had high hsa-miR-100-5p expression levels exhibited worse overall survival (Figure 2D) and disease-free survival (Figure 2E) than those with low hsa-miR-100-5p expression. These findings suggested that hsa-miR-100-5p may play a crucial role in STAD development.

### Selection and Validation of Hsa-miR-100-5p-Related Genes

**FIGURE 3:**
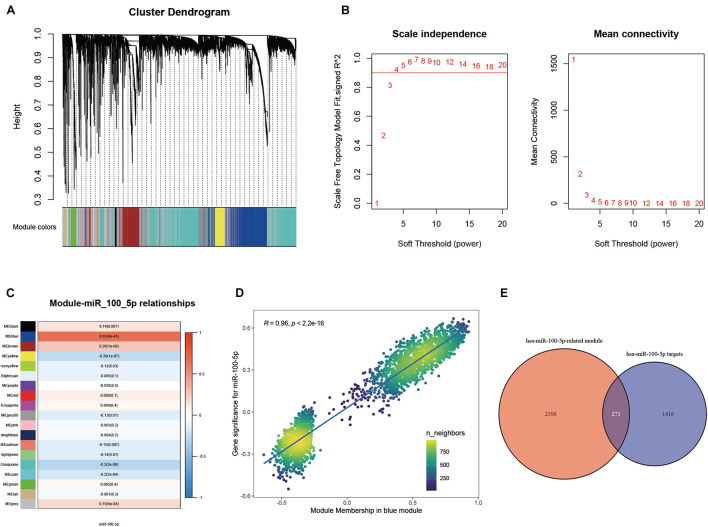
Selection of miR-100-5p-related candidate genes. **(A)** The cluster dendrogram of genes in TCGA-STAD. Each branch in the figure represents one gene; meanwhile, every color below represents one co-expression module. **(B)** Analysis of the scale-free fit index for a variety of soft-thresholding powers. **(C)** The relationships between miR-100-5p expression and various gene modules. **(D)** The correlation between gene significance for miR-100-5p and module membership in the blue module. **(E)** Candidate genes were screened by taking interaction with hsa-miR-100-5p targets and genes in the hsa-miR-100-5p-related module.

A WGCNA was performed with gene expression profiles to establish a scale-free co-expression network. A total of 18 gene modules were generated with the optimal soft threshold set at a power of 4 and the merge cut height set to 0.6 (Figures 3A,B and Supplementary Figure 1A). Among these gene modules, the blue module shows the highest correlation with hsa-miR-100-5p expression and was considered the “hsa-miR-100-5p-related module” (*r* = 0.65, *p* = 8e−43) (Figure 3C). In addition, the relationship between the genes related to hsa-miR-100-5p expression and included in the blue module, which consisted of 2,669 genes, exhibited a highly positive correlation (*R* = 0.96, *p*<2.2e−16) (Figure 3D). By examining the interaction between the “has-miR-100-5p-related module” and potential targets of hsa-miR-100-5p, we found 271 genes considered hsa-miR-100-5p-related candidate genes (Figure 3E).

**FIGURE 4:**
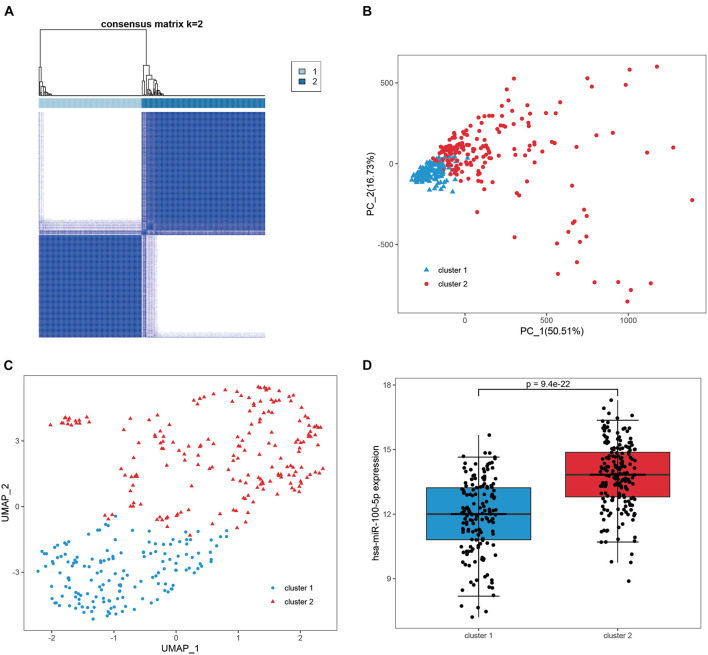
Validation of the candidate genes as robust miR-100-5p-related genes. **(A)** Consensus clustering was utilized to assign 348 samples in TCGA-STAD into two clusters based on the candidate genes. **(B,C)** PCA analysis **(B)** and UMAP analysis **(C)** demonstrated that the two clusters showed absolute dissimilarity. **(D)** The expression levels of hsa-miR-100-5p were significantly different between the two clusters.

We subsequently confirmed that 271 candidate genes were robust hsa-miR-100-5p-related genes based on STAD samples in TCGA, since simultaneous miRNA and gene expression profiles were available for these samples. Consensus clustering was employed to assign 348 samples into two clusters according to the expression of the 271 candidate genes with an optimal *k* of 2 (Figure 4A and Supplementary Figures 1B,C). PCA and UMAP analyses demonstrated that these candidate genes had good discriminating ability, and the two clusters exhibited absolute dissimilarity (Figures 4B,C). Moreover, the expression level of hsa-miR-100-5p between the two clusters was significantly different (*p* = 9.4e−22, Figure 4D). Hence, the 271 candidate genes were considered hsa-miR-100-5p-related genes.

### Construction and Validation of the Hsa-miR-100-5p-Related Prognostic Signature

**FIGURE 5:**
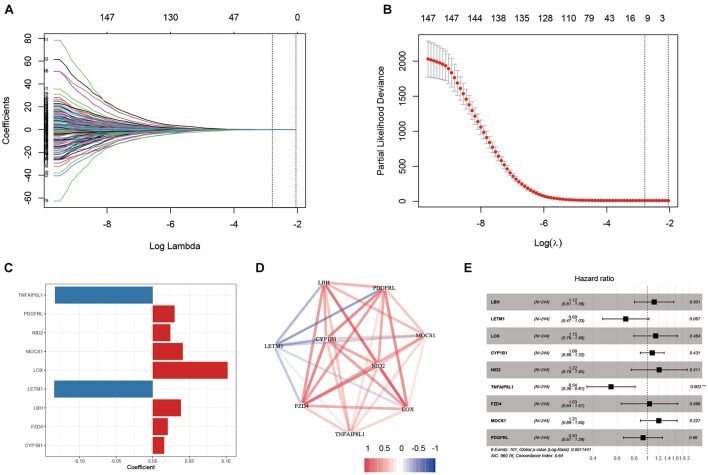
Establishment of an hsa-miR-100-5p-related prognostic signature. **(A)** The changing trajectory of each variable in LASSO Cox regression. **(B)** Selection of the optimal lambda value. **(C)** The coefficients of nine selected genes. **(D)** Co-expression network of selected genes. **(E)** Multivariate Cox analysis of nine selected genes based on the training cohort. **p < 0.01.

**FIGURE 6:**
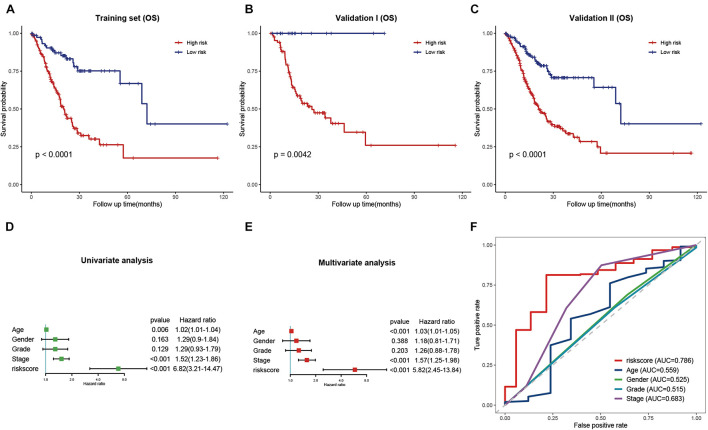
Validation and evaluation of the prognostic signature. **(A–C)** Kaplan–Meier curves of low- and high-risk groups in the training cohort **(A)**, validation cohort **(B)**, and entire cohort **(C)**. **(D,E)** Univariate **(D)** and multivariate **(E)** Cox regression analysis of the risk score and clinical features. **(F)** ROC curves of the risk score and traditional clinical characteristics.

Based on the results obtained with the training cohort, LASSO Cox regression analysis was performed to identify the most robust prognostic genes among the hsa-miR-100-5p-related genes (Figure 5A). A 10-fold cross-validation was subsequently performed to overcome the overfitting effect, and an optimal *λ* value of 0.0613 was selected (Figure 5B). As a result, a panel of nine genes remained on the basis of their individual coefficients (Figure 5C). A correlation network involving the nine genes in the training cohort is shown in Figure 5D. The multivariate Cox regression analysis revealed that TNFAIP8L1 was an independent protective factor for the overall survival of STAD patients (Figure 5E). The hsa-miR-100-5p-related prognostic risk score for each patient in every cohort was calculated using the following formula: risk score = (LBH×0.03841) + (LETM1× −0.13428)+ (LOX × 0.10214) + (CYP1B1 × 0.01567) + (NID2 × 0.02408) + (TNFAIP8L1 × −0.13329) + (FZD4 × 0.02059) + (MOCS1 × 0.04101) + (PDGFRL × 0.02987). Then, patients were assigned into low- and high-risk groups according to the optimal cutoff risk score in each cohort. As shown in Figure 6A, the Kaplan–Meier survival analysis performed on the training cohort data demonstrated that patients in the high-risk group exhibited unfavorable overall survival compared with patients in the low-risk group (*p* < 0.0001). Similarly, in the validation cohort and the entire cohort, patients in the high-risk group exhibited a poor clinical outcome (Figures 6B,C).

### Evaluation of the Prognostic Signature and Clinical Features of Stomach Adenocarcinoma

Univariate and multivariate Cox regression analyses were performed to evaluate whether the risk signature was an independent prognostic factor for STAD. In the univariate Cox regression analysis, the HR of the risk score and the 95% confidence interval (CI) were 6.82 and 3.21–14.47, respectively (*p*<0.001) (Figure 6D). The HR was 5.82, and the 95% CI was 2.45–13.84 in multivariate Cox regression analysis (*p*<0.001) (Figure 6E). These results indicated that the hsa-miR-100-5p-related signature was unrelated to clinical characteristics, including age, sex, tumor grade, and pathological stage. ROC curves were plotted to better evaluate the uniqueness and susceptibility of the prognostic signature in predicting the overall survival of STAD patients. As shown in Figure 6F, the AUC value of the risk score was higher than that of the clinical features, suggesting that the risk score can better predict the prognosis of STAD patients.

### Development and Assessment of a Prognostic Nomogram

**FIGURE 7:**
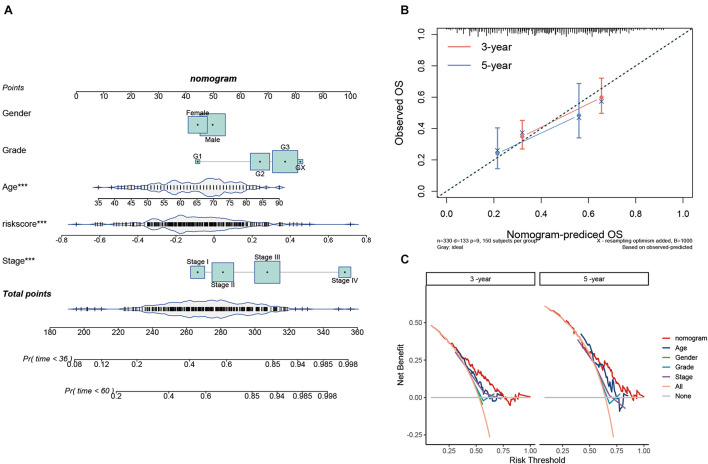
Construction and validation of the prognostic nomogram. **(A)** Nomogram for 3- and 5-year survival predictions. **(B)** Calibration curves for validating the accuracy of the 3- and 5-year survival predictions. **(C)** DCA analyses of the nomogram and clinical features at two different time points.

To provide a quantitative tool for predicting the survival rate of patients with STAD, a nomogram comprising the risk score and clinical characteristics was introduced (Figure 7A). Calibration curves showed that the predicted versus observed rates of 3- and 5-year overall survival exhibited ideal consistency (Figure 7B). In addition, DCA curves graphically illustrated that, at two different time points, the nomogram performed better than clinical features such as age, sex, tumor grade, and pathological stage (Figure 7C). These results indicated that the nomogram exhibited predominant predictive ability and can be used to improve the prognostics of STAD patients.

### Estimation of the Tumor Immune Microenvironment

**FIGURE 8:**
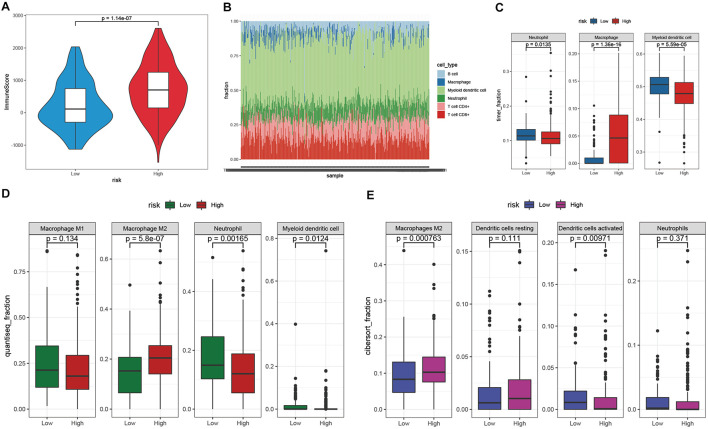
Investigation of the TIME between the low- and high-risk groups. **(A)** The immune score of the high-risk group was higher than that of the low-risk group. **(B)** The landscape of tumor-infiltrating cells of STAD samples. **(C–E)** The fractions of tumor-infiltrating cells between the low- and high-risk groups were estimated by TIMER **(C)**, quanTIseq **(D)**, and CIBERSORT **(E)**.

The TIME is involved in the development and metastasis of cancer, affecting the prognosis of patients (Ren et al., 2018; Melaiu et al., 2019). To investigate the effect of the prognostic signature on the TIME of STAD, we estimated the immune score and tumor-infiltrating cells in the low- and high-risk groups. The high-risk group exhibited a higher immune score than the low-risk group (Figure 8A). Subsequently, the fraction of immune cell types in each sample was evaluated with the TIMER algorithm, and the resulting landscape is displayed in Figure 8B. We compared each cell type between the two groups and found that the fraction of macrophages in the high-risk group was distinctly greater than that in the low-risk group (*p* < 0.001), while the fractions of neutrophils and myeloid dendritic cells showed the opposite trend (Figure 8C). By employing the quanTIseq algorithm, we discovered that the presence of M2 macrophages but not M1 macrophages led to a high immune score in the high-risk group (Figure 8D). To validate our findings, the CIBERSORT algorithm was used, and the results showed that the presence of M2 macrophages led to significant differences in the TIME between the low- and high-risk groups (Figure 8E). Moreover, activated dendritic cells but not resting dendritic cells played an important role in the TIME; however, the fractions of neutrophils were not significantly different between the two groups (Figure 8E). Taken together, the results showed that M2 macrophages and activated dendritic cells play a crucial role in the TIME of STAD.

### Exploration of the Potential Regulatory Mechanisms in the High- and Low-Risk Groups

**FIGURE 9:**
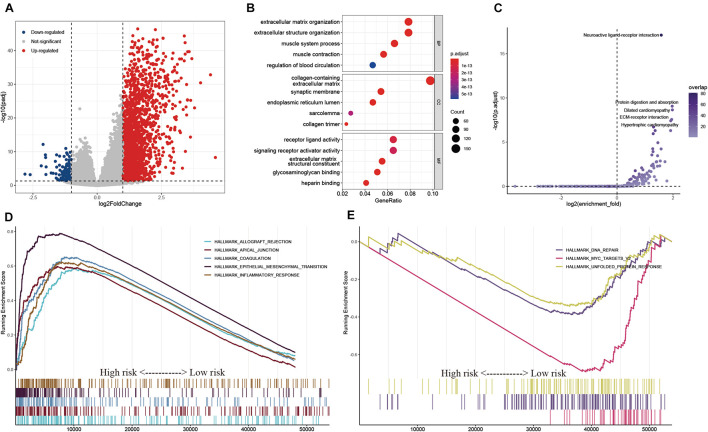
Exploration of the potential regulatory mechanisms resulting in the differences of TIME between the low- and high-risk groups. **(A)** Volcano plot of DEGs between the low- and high-risk groups. **(B,C)** GO **(B)** and KEGG **(C)** enrichment analyses of DEGs. **(D,E)** Hallmarks were significantly enriched in the high-risk **(D)** and low-risk **(E)** groups.

To explore the potential regulatory mechanisms resulting in differences in the TIME between the low- and high-risk groups, the gene expression profiles of these two groups were analyzed. A total of 121 downregulated and 2,337 upregulated genes were identified in the high-risk group, compared with the corresponding gene expression level in the low-risk group (Figure 9A). A functional annotation analysis revealed that these genes are mainly involved in extracellular matrix organization and receptor ligand activity (Figure 9B). A KEGG pathway analysis indicated that the pathways including “neuroactive ligand–receptor interaction” and “ECM–receptor interaction” are significantly enriched with these genes (Figure 9C). By setting the gene signatures in “h.all.v7.4.symbols.gmt” as the reference gene set, a GSEA was performed, and the results showed that hallmarks such as the epithelial–mesenchymal transition and inflammatory response were significantly enriched in the high-risk group, while DNA repair and unfolded protein response were dynamically correlated with the low-risk group (Figures 9D,E).

### Prediction of Cancer Chemotherapy and Immunotherapy

**FIGURE 10:**
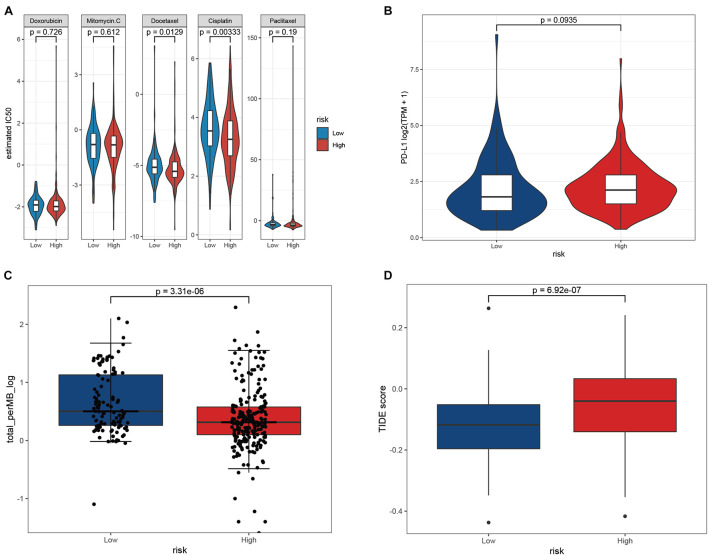
Chemotherapy and immunotherapy sensitivity prediction. **(A)** The estimated IC50 for various chemotherapeutic drugs. **(B–D)** The differences of PD-L1 expression **(B)**, TMB **(C)**, and TIDE scores **(D)** between the low- and high-risk groups.

Chemotherapy drugs, including doxorubicin, mitomycin C, docetaxel, cisplatin, and paclitaxel, have proven to be helpful for gastric cancer treatment, and some of these drugs are in clinical trials (Miranda et al., 2014; Zheng et al., 2017; Bando et al., 2018; Hemati et al., 2019; Yoshida et al., 2019). We compared the differences in the estimated IC50 levels between the low- and high-risk groups. Our data showed that the estimated IC50 values of docetaxel (*p* = 0.013) and cisplatin (*p* = 0.003) were significantly higher in the low-risk group, indicating that patients in the high-risk group were more sensitive to docetaxel and cisplatin chemotherapy (Figure 10A). However, there was no distinct difference in the estimated IC50 levels of the other three drugs. PD-L1 expression level and tumor mutation burden are widely used biomarkers for predicting the response to immunotherapy (Zhu and Lang, 2017; Chan et al., 2019). We observed that there was no significant difference in PD-L1 expression between the low- and high-risk groups, while the level of TMB was distinctly higher in the low-risk group than in the high-risk group (Figures 10B,C). In addition, the TIDE score was introduced to evaluate the responses to ICB therapies, and our data showed that patients with low-risk scores exhibited lower TIDE scores than patients with high-risk scores (Figure 10D) (Jiang et al., 2018). Hence, these findings indicated that patients with high-risk scores may be more sensitive to docetaxel and cisplatin chemotherapy, and patients with low-risk scores may be more likely to benefit from immunotherapy.

### External Validation of the Hsa-miR-100-5p-Related Prognostic Signature and Meta-Analysis

**FIGURE 11:**
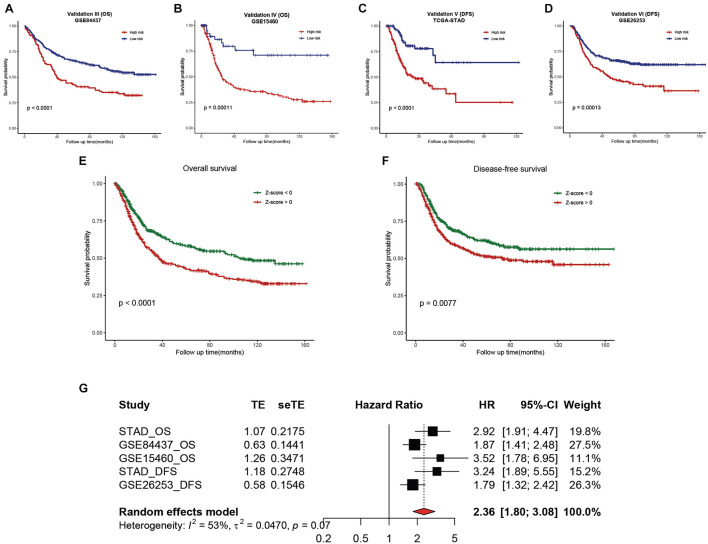
External validation of the established hsa-miR-100-5p-related prognostic signature. **(A,B)** Kaplan–Meier analysis of the low- and high-risk groups on overall survival in GSE84437 **(A)** and GSE15460 **(B)**. **(C,D)** Kaplan–Meier curves of the low- and high-risk groups on disease-free survival in TCGA-STAD **(C)** and GSE26253 **(D)**. **(E,F)** Kaplan–Meier survival analyses on overall survival **(E)** and disease-free survival **(F)** in pooled cohorts. **(G)** Meta-analysis was performed to calculate the pooled HR of the prognostic signature.

To validate the reliability of the hsa-miR-100-5p-related prognostic signature, we used three additional cohorts, namely, the GSE84437, GSE15460, and GSE26253 datasets, as external validation cohorts. The established signature showed good performance in predicting the survival rates of patients not only in terms of overall survival but also disease-free survival (Figures 11A–D). In addition, Kaplan–Meier analysis was performed to evaluate survival differences in the pooled cohorts, and the hsa-miR-100-5p-related signature retained its prognostic capacity to discriminate low- and high-risk subsets with a *Z*-score of zero as the cutoff value (Figures 11E,F). Meta-analysis was performed to calculate the pooled HR and 95% CI of the prognostic signature, and the results were 2.36 and 1.80–3.08, respectively (Figure 11G). These results demonstrated that the hsa-miR-100-5p-related signature was robust.

## Discussion

Recently, studies investigating miRNAs and tumors have attracted widespread attention. Increasing evidence indicates that the aberrant expression of miRNAs is related to certain cancer types (Lee and Dutta, 2009). Hu et al. revealed that miR-532 was overexpressed in gastric cancer tissues and promoted tumor migration (Hu et al., 2017). Setijono et al. found that miR-218 functioned as a tumor suppressor, while miR-129 promoted cancer progression in breast cancer (Setijono et al., 2018). Tang et al. revealed that forced expression of miR-208a introduced upon X-ray irradiation promoted cell proliferation and radioresistance in lung cancer cells (Tang et al., 2016). Stomach Adenocarcinoma is the most prevalent histology in gastric cancer, but the prognosis remains extremely poor. In consideration of the crucial roles of miRNAs in almost all aspects of cancer biology, targeting specific miRNAs may be an efficient strategy for treating cancers, including STAD. Several miRNA-targeted therapeutics have been developed and are undergoing clinical trials, including miR-34, which has been used to treat solid tumors (Rupaimoole and Slack, 2017). Nevertheless, studies on miRNAs in STAD remain limited, and further research is necessary. In the present study, we found that hsa-miR-100-5p was the only miRNA abnormally expressed in STAD samples and correlated with the prognosis of STAD patients. Inspired by these findings, we established a miR-100-5p-related prognostic signature to aid in the clinical management of patients with STAD.

In our study, a WGCNA was performed with the aim of selecting a miR-100-5p-related gene module. By analyzing the overlap of miR-100-5p targets and genes in a miR-100-5p-related module, candidate genes were identified and subsequently validated through PCA and UMAP. We performed LASSO Cox regression analysis based on the training cohort and miR-100-5p-related genes. Ultimately, a total of nine prognostic genes were chosen on the basis of their individual coefficients. Among these genes, LBH promotes angiogenesis in glioma through VEGFA-mediated ERK signaling under hypoxic conditions (Jiang et al., 2019). LETM1 contributes to cancer cell proliferation and invasion *via* the PI3K/Akt signaling pathway and has been identified as a potential biomarker to predict the prognosis of gastric cancer and lung cancer (Piao et al., 2019; Li et al., 2020; Zhang et al., 2020). Additionally, LOX is considered to be a potential relapse marker for pancreatic cancer patients (Ma et al., 2019). CYP1B1, a member of the CYP superfamily, plays a critical role in oxidative metabolism and promotes the development of breast cancer (Hwang et al., 2019). Previous studies have revealed that NID2 is overexpressed in gastric cancer and can boost gastric cancer cell invasion (Yu et al., 2019). TNFAIP8 L1, an independent protective factor for STAD patients identified through multivariate Cox analysis, suppresses invasion and migration by downregulating the Wnt/beta-catenin pathway in gastric cancer (Liu et al., 2018). Chen et al. demonstrated that FZD4 is a novel target of miR-101 in bladder cancer cells (Chen et al., 2019). PDGFRL, which is regarded as a tumor-suppressor gene, inhibits the proliferation and invasion of colorectal cancer cells *in vitro* (Guo et al., 2010).

Subsequently, STAD patients were assigned into low- and high-risk groups according to the risk score calculated by the established miR-100-5p-related prognostic signature. Patients with high-risk scores exhibited poor clinical outcomes. Univariate and multivariate Cox analyses revealed that risk score was an independent prognostic risk factor for patients with STAD. ROC analysis demonstrated that risk score was superior to other clinical characteristics in predicting the overall survival of STAD patients. Then, a nomogram of integrated risk score and clinical features was introduced and exhibited acceptable consistency between the predicted and observed rates for 3- and 5-year overall survival. Moreover, a DCA analysis revealed that the nomogram was superior in determining survival than traditional clinical features.

The TIME is known to foster tumor growth and metastasis. Targeting the main components and regulatory mechanism of the TIME would improve the anticancer immune response and immunotherapy treatment (Pitt et al., 2016). Therefore, we investigated the TIME of STAD using a step-by-step approach. The immune score in the high-risk group was distinctly greater than that in the low-risk group. Several deconvolution algorithms were employed to estimate the tumor-infiltrating immune cells, and the results showed that M2 macrophages accounted for the difference in the TIME between the two groups. Previous studies have revealed that M2-polarized tumor-associated macrophages (TAMs) release a variety of anti-inflammatory cytokines and chemokines that suppress dendritic cell maturation, limiting antigen presentation (Ruffell et al., 2014; Vitale et al., 2019). We also observed that the fraction of activated dendritic cells in the high-risk group was significantly lower than that in the low-risk group, which agrees with the research of Ruffell et al. (Ruffell et al., 2014). In addition, M2-like TAMs prevent tumor infiltration by cytotoxic T cells, which may be associated with the worse clinical outcome of STAD patients in the high-risk group (Vitale et al., 2019). To explore the potential regulatory mechanism resulting in the difference in the TIME between the two groups, several functional enrichment analyses were performed. Differentially expressed genes (DEGs) were mainly involved in extracellular matrix–receptor interactions and related biological processes. A GSEA revealed that genes involved with the epithelial–mesenchymal transition and inflammatory responses were significantly enriched in the high-risk group. The epithelial–mesenchymal transition is involved in tumorigenesis and confers metastatic properties onto cancer cells, promoting the invasion of tumors (Mittal, 2018). Inflammation is considered one of the characteristics of cancer development and reduces the rate of survival and quality of life. Taken together, these findings on the TIME and potential regulatory mechanisms may explain the poor prognosis of patients in the high-risk group.

Finally, we evaluated the sensitivity of patients to chemotherapy and immunotherapy. The results showed that patients with higher risk scores may be more sensitive to docetaxel and cisplatin chemotherapy. A low-risk score was correlated with a high TMB and low TIDE score, which indicated that patients in the low-risk group may receive greater benefit from immunotherapy. Although chemotherapy and immunotherapy provide variably effective treatments of human cancer, the therapeutic outcome is not satisfactory because of increasing resistance and a lack of biomarkers (Holohan et al., 2013; O’Donnell et al., 2019). Our results may assist in the development of more individualized therapeutic strategies for treating STAD. Finally, external cohorts were used to validate the robustness of the established miR-100-5p-related prognostic signature. We are also aware of the limitations of this study. The number of patients in the training cohort was slightly fewer than ideal, and this study could have been improved if had we merged some cohorts and removed batch effects. In addition, the results of this study would be more convincing with experimental validation. In addition, the molecular mechanisms of the genes in the prognostic signature need to be further investigated.

## Conclusion

In conclusion, we identified the abnormal expression and prognostic value of hsa-miR-100-5p in STAD. In addition, we constructed an hsa-miR-100-5p-related prognostic signature that performed well in predicting sensitivity to chemotherapy and immunotherapy and can be used to improve prognostic risk stratification for STAD patients. Our study may aid in the development of more individualized therapeutic strategies and improve the clinical outcome of STAD patients.

## Data Availability Statement

The datasets presented in this study can be found in online repositories. The names of the repository/repositories and accession number(s) can be found in the article/Supplementary Material.

## Author Contributions

GW and LY designed the study, analyzed the data, and wrote the manuscript. XJ and RC provided funding acquisition. MH, RH, and YW performed the experiments and analyzed the data. BC, XJ, and RC supervised the research, analyzed the data, and wrote the manuscript. All authors read and approved the final submitted manuscript.

## Conflict of Interest

The authors declare that the research was conducted in the absence of any commercial or financial relationships that could be construed as a potential conflict of interest.

## Publisher’s Note

All claims expressed in this article are solely those of the authors and do not necessarily represent those of their affiliated organizations, or those of the publisher, the editors and the reviewers. Any product that may be evaluated in this article, or claim that may be made by its manufacturer, is not guaranteed or endorsed by the publisher.

## Footnotes

1
https://portal.gdc.cancer.gov/

2
https://www.ncbi.nlm.nih.gov/geo/

3
https://www.gsea-msigdb.org/gsea/index.jsp

## Supplementary Material

Supplementary Figure 1 **(A)** The relationships between different gene modules. **(B)** Relative change in area under the cumulative distribution function (CDF) curve for *k* = 2 to 6. **(C)** Tracking plot for *k* = 2 to 6.

## Abbreviations

AUC: area under the curve
BP: biological process
CC: cellular component
CI: confidence interval
CIBERSORT: cell-type identification by estimating relative subsets of RNA transcripts
DEMs: differentially expressed miRNAs
DFS: disease-free survival
ESTIMATE: Estimation of STromal and Immune cells in MAlignant Tumors using Expression data
GEO: Gene Expression Omnibus
GO: Gene Ontology
GSEA: gene set enrichment analysis
HR: hazard ratio
IC50: the half maximal inhibitory concentration
ICB: immune checkpoint blockade
KEGG: Kyoto Encyclopedia of Genes and Genomes
LASSO: least absolute shrinkage and selection operator
MF: molecular function
OS: overall survival
PCA: principal component analysis
ROC: receiver operating characteristic curve
STAD: stomach adenocarcinoma
TAMs: tumor-associated macrophages
TCGA: The Cancer Genome Atlas
TIME: tumor immune microenvironment
TIMER: Tumor Immune Estimation Resource
TMB: tumor mutation burden
UMAP: uniform manifold approximation and projection
WGCNA: weighted gene co-expression network analysis.
