# Supplementary figures and images for: Comprehensive Analysis of the Prognostic Significance of Hsa-miR-100-5p and Its Related Gene Signature in Stomach Adenocarcinoma
Source: Front Cell Dev Biol. 2021 Sep 17;9:736274. doi: 10.3389/fcell.2021.736274 (PMC8484799; doi:10.3389/fcell.2021.736274)

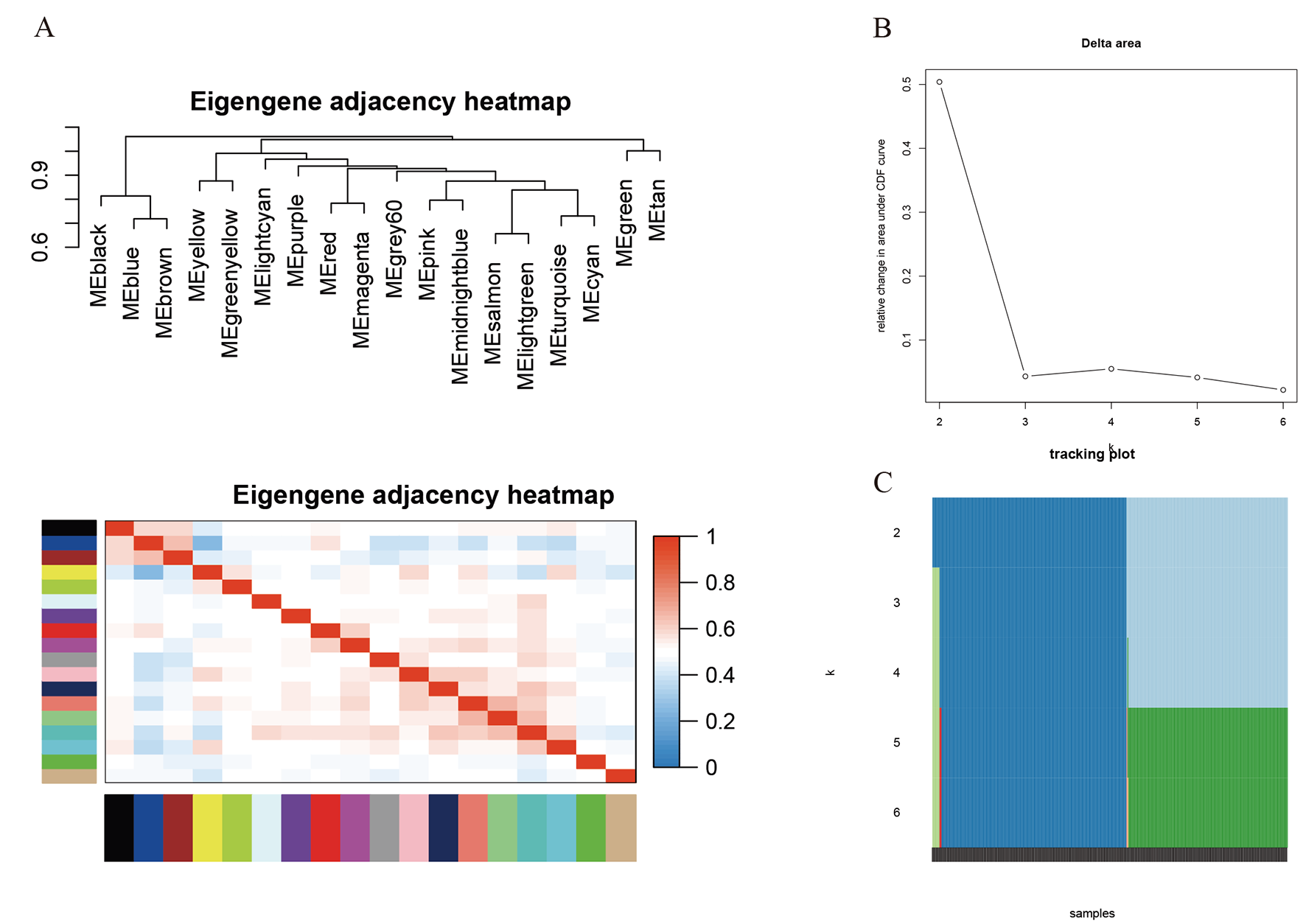

Supplement: Supplementary Figure 1 — (A) The relationships between different gene modules. (B) Relative change in area under the cumulative distribution function (CDF) curve for k = 2 to 6. (C) Tracking plot for k = 2 to 6. [file Image_1.TIF]
